# SIRT1 deacetylates mitochondrial trifunctional enzyme α subunit to inhibit ubiquitylation and decrease insulin resistance

**DOI:** 10.1038/s41419-020-03012-9

**Published:** 2020-10-02

**Authors:** Yan-Mei Wang, Ting-Lei Huang, Chao Meng, Jia Zhang, Ning-Yuan Fang

**Affiliations:** 1 Department of Geriatrics, Ren Ji Hospital, School of Medicine, Shanghai Jiao Tong University, No. 160 Pujian Road, Pudong New Area, Shanghai 200127 China; 2 Department of Oncology, Ren Ji Hospital, School of Medicine, Shanghai Jiao Tong University, No. 160 Pujian Road, Pudong New Area, Shanghai 200127 China

**Keywords:** Drug screening, Metabolic syndrome

## Abstract

Dysregulation of free acid metabolism is a major contributor to the development of insulin resistance and diabetes. Mitochondrial trifunctional enzyme subunit (MTPα) has a critical role in fatty acid β-oxidation. However, the association between MTPα and insulin resistance is not definitively known. Here, we aimed to determine how MTPα affects insulin resistance. We tested how MTPα affected glucose uptake in insulin-resistant 3T3-L1 adipocytes and white adipose tissue (WAT) of db/db diabetic mice. We also measured how acetylation and ubiquitylation modifications regulated MTPα activation and stability, using quantitative real-time polymerase chain reactions, immunoblotting, and immunoprecipitation. We found that MTPα overexpression promoted glucose uptake via Glut4 translocation to the plasma membrane in 3T3-L1 adipocytes. Moreover, MTPα upregulation decreased glycemia in db/db mice. Deacetylation increased MTPα protein stability and its ability to reduce insulin resistance. The activation of SIRT1, a major deacetylase, prevented MTPα degradation by decreasing its acetylation in adipocytes. Our study demonstrates a new role for MTPα in reducing insulin resistance. Acetylation and ubiquitylation modifications of MTPα were crucial to regulating its function in glucose metabolism.

## Introduction

Insulin resistance occurs when a given concentration of insulin produces less than the expected effect on target cells, which can lead to impaired glucose intolerance^1^. Insulin resistance can occur with obesity, pregnancy, burn trauma, and metabolic syndrome, and can cause type 2 diabetes mellitus (T2DM) and cardiovascular dysfunction. Although many researchers study the pathogenesis of insulin resistance^2^, little is known about the underlying mechanism that initiates and advances insulin resistance.

Dysregulation of free acid metabolism is a major contributor to insulin resistance and T2DM^3,4^. An increase in free fatty acids is associated with defective fatty acid oxidation, which induces or aggravates insulin resistance in adipose, liver, and muscle tissue by directly or indirectly generating metabolites and altering insulin signaling. Free fatty acid reduction is a target for treating insulin resistance^5^.

Mitochondrial trifunctional protein α-subunit (MTPα) is involved in fatty acid β-oxidation (FAO). MTPα has long-chain hydratase activity, which catalyzes the second step of fatty acid β-oxidation, as well as 3-hydroxyacy-CoA dehydrogenase activity, which catalyzes the third step. A MTPα gene defect causes defective mitochondrial fatty acid oxidation and reverses insulin-stimulated suppression of hepatic glucose production^6^. Furthermore, heterozygous mice lacking MTPα have significantly reduced fatty acid oxidation in liver tissue and develop hepatic steatosis and insulin resistance^7^. Thus, MTPα is a critical enzyme in fatty acid β-oxidation and may play an important role in insulin signaling. However, there is no definitive association between MTPα and insulin resistance.

During diabetes or insulin resistance, excess free fatty acids and high glucose concentrations increase the concentration of TCA cycle products, such as acetyl-CoA and NADH^8,9^. Increased acetyl-CoA and NADH concentrations induce acetylation of mitochondrial proteins, including MTPα^10,11^. Lysine acetylation is a common protein post-translational modification that regulates cellular metabolism^12,13^. MTPα can be acetylated on three lysine residues 350/383/406, which affects its function in hepatic steatosis^14^. However, the relationship between MTPα acetylation and insulin resistance is unclear.

SIRT1 is an NAD^+^-dependent deacetylation enzyme. SIRT1 regulates glucose metabolism through its deacetylase activity^15^ and directly or indirectly contributes to insulin signaling^16^. However, elevated glucose levels in T2DM downregulate the protein expression of SIRT1, leading to increased MTPα acetylation. Further, the direct role of SIRT1 in regulating MTPα acetylation remains unknown.

In this study, we demonstrated a novel function for MTPα in reducing insulin resistance. MTPα overexpression promoted insulin-dependent glucose uptake and activated the insulin signaling pathway. We identified MTPα acetylation at four lysine sites (K359, K383, K620, and K625). Mutation of the K625 acetylation site blocked MTPα ubiquitylation and its subsequent degradation. SIRT1 deacetylated MTPα at the K625 site and repressed its degradation. MTPα deacetylation mediated by 9-PAHSA or resveratrol, a SIRT1 agonist, decreased insulin resistance.

## Materials and methods

### Cell culture and cell differentiation

3T3-L1 preadipocytes were obtained from the Chinese Academy of Medical Sciences and Peking Union Medical College (Beijing, China). 3T3-L1 preadipocytes were grown in DMEM supplemented with 10% FBS, 200 U/ml penicillin and 200 U/ml streptomycin in 5% CO_2_ humidified atmosphere at 37 °C until confluence. Two days after confluence, to induce adipocyte differentiation, cells were incubated for 48 h in DMEM supplemented with 10% FBS containing 500 μM 3-isobutyl-1-methylxanthine (IBMX), 0.25 μM dexamethasone, and 5 μg/ml insulin. Then the cells were maintained in culture medium supplemented with insulin only, which were changed every 2 days until establishment of insulin resistance.

The SIRT1 activator resveratrol was added to the mature adipocytes during the period of induction of insulin resistance at the final concentration of 10 µM, while the SIRT1-specific inhibitor EX527 was added at the final concentration of 10 µM. 9-PAHSA was added at the final concentration of 20 µM. The proteasomal inhibitor MG132 was added to the mature adipocytes 4 h before harvest at the final concentration of 10 µM. For protein stability assay, cells were treated with 100 μg/ml Cycloheximide (CHX, from Sigma) for the indicated time before harvest.

### Induction of insulin resistance^2^

Treatment with recombinant mouse TNF-α (4 ng/ml) was initiated with mature adipocytes from day 8 of differentiation. Media was changed daily for TNF-α treatment for a total incubation time of 4 days.

### Animal studies

All animal experiments were approved by Fudan University Animal Care and Use Committee and also meet the guidelines of the National Institutes of Health Guide for the Care and Use of Laboratory Animals (UJS-LAER-2017042301). Eight to 12-week-old Male C57BL6/J mice, db/db mice and their control littermates were purchased from the Model Animal Research Center of Nanjing University (Nanjing, China). At 8 weeks of age, the C57BL/6 mice were given high fat diet (HFD, Shanghai SLAC Company) for 2 months, with normal chow diet mice as control. Mice with random blood glucose >11.1 mmol/L were considered as insulin-resistant mice. All mice were housed in colony cages with ad libitum access to food and water. Mice were kept on a 12/12 h light/dark cycle in a temperature-controlled environment.

Db/db mice were divided randomly into two groups: control db/db group and db/db plus 9-PAHSA group (50 mg/kg per day). 9-PAHSA (0.2 ml) was given by gavage once per day for 10 days. The control mice were given the same volume of vehicle (50% PEG 400, 0.5% Tween 80, 49.5% H2O).

Each test group included five mice. All mice were euthanized by intraperitoneal injection of 60 mg/kg sodium pentobarbital. Then abdominal adipose tissue was removed for further study.

### Measurement of random glycemia in db/db mice

Blood was collected from the tail vein of each mouse using heparin-coated capillary tubes. Glucose levels were determined using Accu-Check active bands (Roche Diagnostics).

### Gene expression analysis

RNA isolation, reverse transcription, and PCR were performed as described previously^17^. Briefly, trizol reagent was utilized to extract total RNA from 3T3-L1 adipocytes. Then, 1 μg of total RNA was subjected to reverse transcription using the PrimeScript™ RT Reagent kit. Gene expression was evaluated by Quantitative real-time PCR (RT-PCR) analysis using SYBR Green reagents (SYBR® Premix Ex Taq™) and the LightCycler® 480 Real-Time PCR System (Roche Diagnostics, Basel, Switzerland). RT-PCR reactionshad a final volume of 10 μl. The following cycler program was used for reactions: initial denaturing at 95 °C for 5 min, followed by 45 cycles of denaturing at 95 °C for 10 s and annealing and extension at 60 °C for 20 s. The threshold cycle (Ct) value was computed for each amplification curve, and ΔCt values were calculated by subtracting the Ct value for β-actin RNA from the Ct value for each experimental sample. The results were expressed as fold-changes with respect to the control using the 2-ΔΔCT formula. The primer sequences for qPCR were as follows: MTPα, 5′-ACA TCG GAG CTG TCT TTG GG-3′ (forward) and 5′-GAC TCG TAC TTC CGT AGC CG-3′ (reverse); β-actin, 5′-AGC CTT GTA GGT ACC CAA CC-3′ (forward) and 5′-TCC CAC TCA CCT GAG GTG CTG AA-3′ (reverse).

### Glucose uptake assay

Glucose uptake assay in 3T3-L1 adipocytes were performed as described previously^18^. Briefly, cells in 96 well dishes were washed twice with PBS and incubated with 100 μl KRPH/2% BSA for 40 min. Prepare sample background controls, insulin stimulated cells and non-stimulated control samples. (1) Sample background control (untreated) cells: Do not add insulin and 2-deoxyglucose (2-DG). (2) Insulin stimulated cells: KRPH/ 2% BSA contained with 10 μM insulin for 20 min and add 10 μl of 10 mM 2-DG for 20 min. (3) Non-stimulated control samples: Non-insulin stimulated cells, but add 10 μl of 10 mM 2-DG for 20 min. Prepare Reaction Mix A and add in all samples. And incubate for 1 hour. Add 90 μl Extraction buffer in each well and heat at 90 °C for 40 min. Prepare Reaction Mix B fresh and add 38 μl in all wells. Measure output OD at 412 nm wavelength on a microplate reader in a kinetic mode, every 2–3 min, at 37 °C protected from light.

### Immunofluorescence staining

Immunofluorescence staining for the Glut4 membrane translocation analysis was conducted as described previously^18^. Briefly, cells were blocked with 5% BSA for 30 min at room temperature with membrane rupture treatment by Triton to detect total Glut4 or without membrane rupture to determine membrane distribution. Cells were incubated at 4 °C with anti-Glut4 antibody overnight. Equal PBS was added instead of Glut4 as a negative control. The Cy3-conjugated secondary antibody was applied to the samples at room temperature for 1 h. After washing with PBS, images were immediately captured under an immunofluorescence microscope.

To investigate the subcellular localization of SIRT1 in 3T3-L1 adipocytes, adipocytes were stained with MitoTracker (Molecular Probes), together with specific antibody for SIRT1 detection. Images were captured under an immunofluorescence microscope.

### Immunoprecipitation (IP)

IP was performed in lysates prepared from 3T3-L1 adipocytes (100 μg total protein) using either the acety-Lys antibody, Ub antibody or normal rabbit IgG at 4 °C overnight. On the next morning, the protein-antibody complex was incubated with 15 μl magnetic protein A + G beads for 1 h at 4 °C with gentle rotation. The antibody-protein-bead complexes then were washed three times with IP buffer. The protein in the complex then was eluted with 30 μl 1× loading buffer and boiled before running on a 12% SDS-polyacrylamide gel. The proteins were transferred to nitrocellulose membranes, and acety-Lys/Ub-associated MTPα proteins were immunoblotted using antibodies against MTPα.

### RNA interference

For MTP knockdown induction in vitro, the 3T3-L1 preadipocytes were transfected with control shRNA (shCON) or MTPα-targeted shRNA (shMTPα) lentiviral particles. The target sequence used against mouse MTPα was as follows: 5′-TCTCCCAATCAATCAAATT-3′; and the sequence of the control shRNA was as follows: 5′-TTCTCCGAACGTGTCACGT-3′. For MTPα overexpression induction in vitro, 3T3-L1 preadipocytes were transduced with lentiviral vector containing an MTPα (ovMTPα) expression cassette. Control cells were transfected with the control vector (ovCON). For SIRT1 knockdown induction in vitro, the 3T3-L1 preadipocytes were transfected with control shRNA (shCON) or SIRT1-targeted shRNA (shSIRT1) lentiviral particles. The target sequence used against mouse SIRT1 was as follows: 5′-CCCTCAAGCCATGTTTGAT-3′; and the sequence of the control shRNA was as follows: 5′-TTCTCCGAACGTGTCACGT-3′. For the mutation of MTP acetylation sites induction in vitro, 3T3-L1 preadipocytes were transduced with lentiviral vector containing each lysine mutation to an arginine (R) (K359R, K383R, K620R, K625R). The lentiviral vector and particles were constructed and synthesized by GeneChem (Shanghai, China).

Lentiviral-transfected 3T3-L1 preadipocytes were induced to differentiate into mature adipocytes and then used as cell model throughout the experiments.

### SIRT3 siRNA knockdown in adipocytes

3T3-L1 adipocytes transfected with non-targeting siRNA or SIRT3-targeting siRNA (GeneChem, Shanghai, China) using the Lipo3000 transfection reagent following the manufacturer’s instructions (Life Technologies, Carlsbad, CA, USA). The knockdown efficiency of SIRT3-targeting siRNA was evaluated by western blotting 72 h post-transfection. siRNA sequences are as follows: 5′- CAGCUUGUCUGAAGCAGUATT-3′ and the sequence of the control siRNA was as follows: 5′-UUCUCCGAACGUGUCACGUTT-3′. SIRT3 siRNA-transfected 3T3-L1 adipocytes were treated with10 µM resveratrol for 4 days.

### Mass spectrometry analysis

The peptide samples were analyzed on Thermo Fisher LTQ Obitrap ETD mass spectrometry, Briefly, loaded sample onto an HPLC chromatography system named Thermo Fisher Easy-nLC 1000 equipped with a C18 colume (1.8 mm, 0.15 × 1.00 mm). Solvent A contained 0.1% formic acid and solvent B contained 100% acetonitrile. The elution gradient was from 4 to 18% in 182 min, 18 to 90% in 13 min solvent B at a flow rate of 300 nl/min. Mass spectrometry analysis were carried out carried out at the AIMS Scientific Co.,Ltd.(Shanghai, China) in the positive-ion mode with an automated data-dependent MS/MS analysis with full scans (350–1600 m/z) acquired using FTMS at a mass resolution of 30,000 and the ten most intense precursor ions were selected for MS/MS. The MS/MS was acquired using higher-energy collision dissociation at 35% collision energy at a mass resolution of 15,000.

### SIRT1 deacetylase activity assay

SIRT1 deacetylase activity was assessed using a commercial fluorometric assay kit (cat. no. CS1040; Sigma-Aldrich). 9-PAHSA (100 nmol, 200 nmol, 400 nmol) were incubated with the SIRT1 enzyme, SIRT1 substrate and NAD+ to screen the activator of SIRT1. Moreover, an inhibitor (nicotinamide) and an activator (resveratrol) as negative and positive controls, respectively. Protein (100 µg) were extracted from 3T3-L1 adipocytes or adipose tissue in db/db mice to detect SIRT1 deacetylase activity. The fluorescence emitted, due to deacetylation of the substrate by SIRT1, was measured at 350 nm excitation and 450 nm emission wavelengths using a fluorescence microplate reader (SpectraMax® M5; Molecular Devices, LLC, Sunnyvale, CA, USA).

### Isolation of mitochondria from 3T3-L1 adipocytes

Isolation of mitochondria from adipocytes was performed using Mitochondria Isolation Kit (Sigma-Aldrich). Enriched fractions of mitochondria from adipocytes were purified also according to the protocol from this kit. The possible contamination of mitochondria with nuclei components was excluded by carrying out western blot analysis of H2B, a much abundant nuclear protein. The effective mitochondria isolation was assessed by anti-VDAC1.

### Protein preparation and western blotting analysis

Total protein was extracted with RIPA buffer containing phenylmethylsulfonyl fluoride (PMSF) and Halt Protease and Phosphatase Inhibitor Cocktail. Membrane protein was extracted by membrane and cytosol protein extraction kit (Beyotime Biotechnology, China). The concentration of proteins was tested using the bicinchoninic acid (BCA) protein assay. Protein samples (30 μg) were separated by SDS-PAGE and then transferred to nitrocellulose membranes (Bio-Rad, Richmond, CA, USA). Membranes were blocked in 5% non-fat milk in Tris-buffered saline containing 0.05% Tween-20 (TBST) for 1 h at room temperature. Then, membranes were incubated with primary antibody at 4 °C overnight. Anti-MTPα, anti-IRS1/P-IRS1, anti-Glut4, anti-β-actin, anti-GAPDH, anti-SIRT1, anti-Ace, anti-Ub and anti- Na^+^-ATPase α-1 were from Abcam. Anti-Akt/P-Akt, anti-VDAC1, anti-H2B and secondary antibody were from Cell Signaling Technology. Probed membranes were washed several times with TBST, and then incubated with horseradish peroxidase conjugated secondary antibodies at room temperature for 1 h. Bound antibody was detected with enhanced chemiluminescence (Millipore, Billerica, MA, USA). Protein expression was quantified using Image J software (NIH, USA). Total protein expression was normalized with respect to β-actin/GAPDH expression and membrane protein was normalized with respect to Na^+^-ATPase α-1 expression.

### Statistical analysis

All experiments were repeated at least three times. All data were analyzed using GraphPad Prism software (GraphPad Software Inc., CA, USA) and expressed as the mean ± standard error (SE). One-way ANOVA and two-way ANOVA were used to compare differences among multiple groups, and the non-paired *t*-test was used to analyze two groups after homogeneity of variance testing. A value of *p* < 0.05 was considered statistically significant.

## Results

### Establishment of insulin resistant cell model

**Fig. 1:**
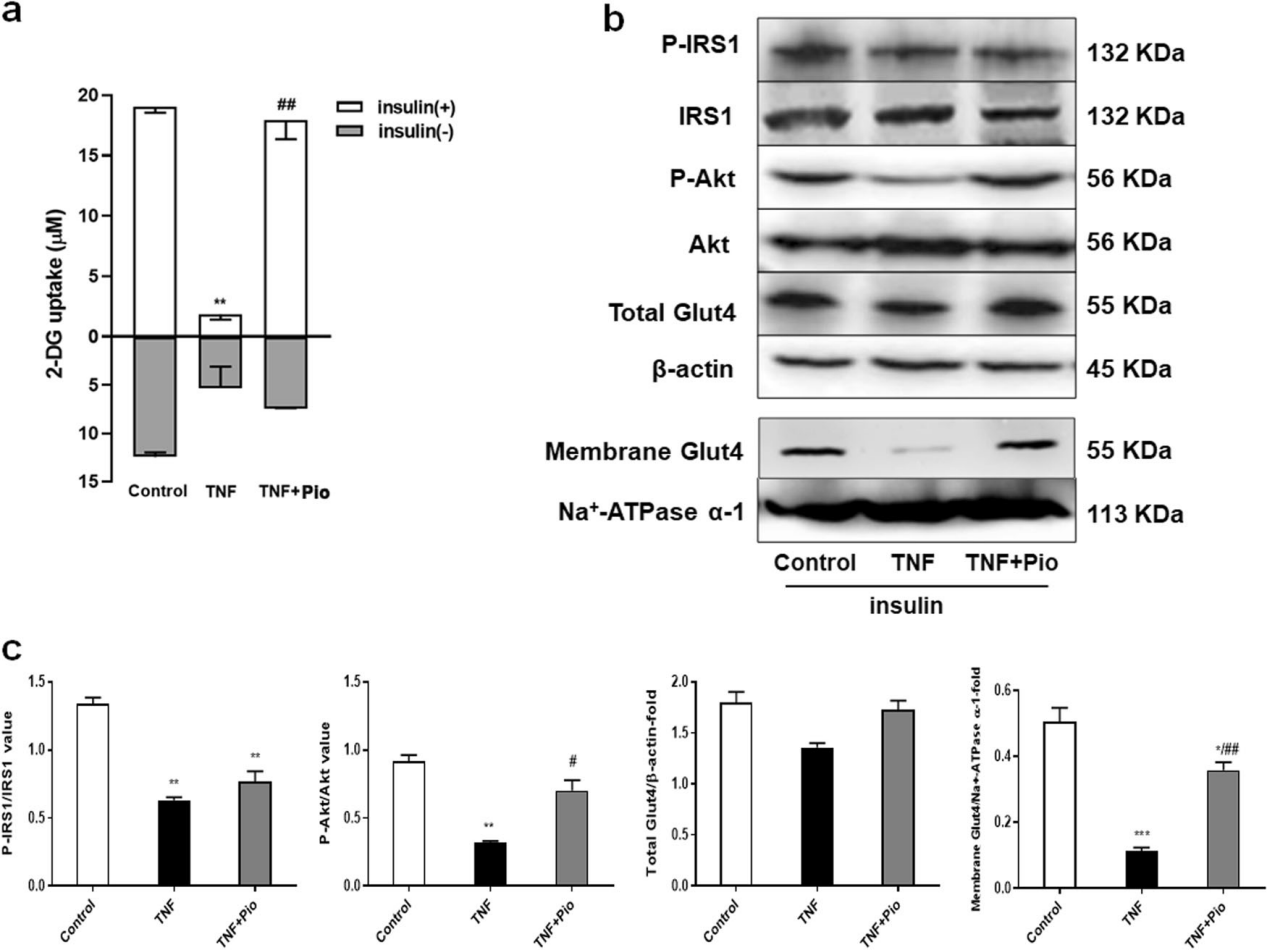
Establishment of insulin resistant state in 3T3-L1 adipocytes. **a** Glucose uptake in 3T3-L1 adipocytes. Basal glucose transport (gray) and insulin stimulated glucose uptake (white) are shown. Adipocytes were untreated, treated with TNF-α (4 ng/ml) alone or TNF-α (4 ng/ml) plus pioglitazone (2 μM) for 4 days. **p < 0.01 vs. insulin alone, ^#^*p* < 0.05 vs. TNF-α plus insulin (one-way ANOVA). **b** Insulin signaling parameters were examined by western blotting analysis in 3T3-L1 adipocytes. Adipocytes were untreated, treated with TNF-α (4 ng/ml) alone or TNF-α (4 ng/ml) plus pioglitazone (2 μM) for 4 days. All adipocytes were treated with insulin (5 μg/ml) for 4 days. **c** Quantification of western blotting. *p < 0.05, **p < 0.01, ***p < 0.001 vs. control, ^#^*p* < 0.05, ^##^*p* < 0.01 vs. TNF-α (one-way ANOVA). Data are representative of at least three different experiments. All data represent means ± standard error (SE).

First, we treated 3T3-L1 adipocytes with TNF-α (4 ng/ml) to create an insulin resistance (IR) model. We assessed the model by measuring how much insulin was needed to stimulate glucose uptake. TNF-α decreased insulin-dependent glucose uptake. However, the decrease was rescued by pioglitazone, a member of the thiazolidinedione (TZD) class of insulin-sensitizing drugs (Fig. 1a).

We also examined how TNF-α affected insulin signaling. IRS1 and Akt activation are major phosphorylation-dependent signaling intermediates in insulin sensitivity. TNF-α treatment decreased insulin-stimulated IRS1 phosphorylation at Thr896 and Akt phosphorylation at Ser473; in contrast, co-treatment with pioglitazone largely prevented TNF-α effects on Akt phosphorylation, while did not reverse TNF-α-inhibited IRS-1 phosphorylation (Fig. 1b, c). TNF-α alone or together with pioglitazone had no effect on total Glut4 levels. However, the expression levels of Glut4 on cell surface were decreased in TNF-α treated 3T3-L1 adipocytes compared with control based on western blotting detection. In contrast, pioglitazone treatment significantly increased membrane Glut4 levels, suggesting that pioglitazone promoted insulin-stimulated Glut4 plasma membrane translocation (Fig. 1b, c). Altogether, these results indicated that we successfully established the IR model in 3T3-L1 adipocytes.

### MTPα increased glucose uptake and activated insulin signaling pathway in TNF-α-induced insulin-resistant 3T3-L1 adipocytes

**Fig. 2:**
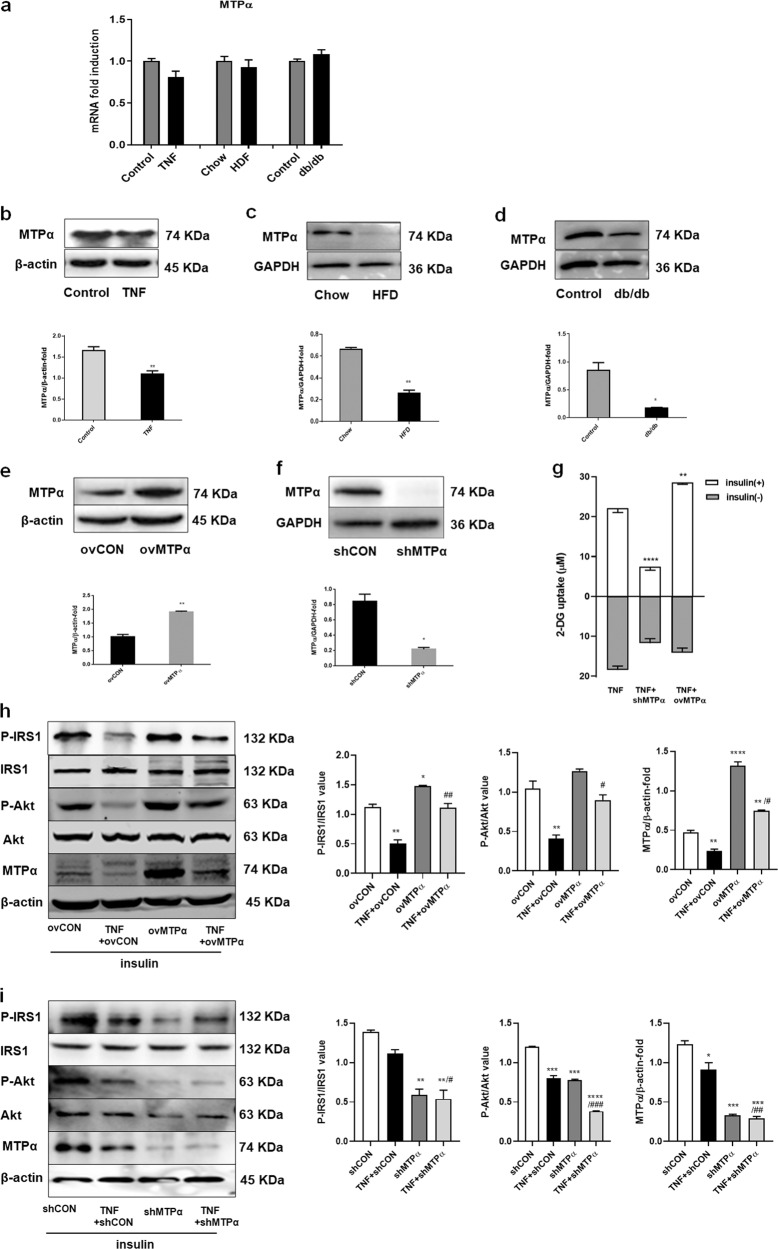
The effect of MTPα on insulin resistance. **a** MTPα mRNA expression in TNF-α (4 ng/ml) treated 3T3-L1 adipocytes, WAT of HFD mice and db/db mice. **b** MTPα protein level in TNF-α (4 ng/ml) treated 3T3-L1 adipocytes. **p < 0.05 vs. control (*t*-test). **c** MTPα protein level in WAT of HFD-induced insulin-resistant mice. **p < 0.01 vs. chow fed mice (*t*-test). **d** MTPα protein level in WAT of db/db diabetic mice. *p < 0.05 vs. control mice (*t*-test). **e** MTPα protein levels as determined by western blotting in 3T3-L1 preadipocytes transfected with ovMTPα. **p < 0.01 vs. ovCON (*t*-test). **f** MTPα protein levels as determined by western blotting using 3T3-L1 preadipocytes transfected with shMTPα. *p < 0.05 vs. shCON (*t*-test). **g** Glucose transport in TNF-α treated 3T3-L1 adipocytes transfected with shMTPα or ovMTPα. Basal glucose transport (gray) and insulin stimulated glucose uptake (white) are shown. **p < 0.01, ****p < 0.0001 vs. TNF-α plus insulin (one-way ANOVA). **h** Insulin signaling examined by western blotting analysis in 3T3-L1 adipocytes. Cells treated with TNF-α (4 ng/ml) alone or together with ovMTPα for 4 days. Meanwhile all adipocytes were treated with insulin (5 μg/ml) for 4 days. *p < 0.05, **p < 0.01, ****p < 0.0001 vs. ovCON; ^#^*p* < 0.05, ^##^*p* < 0.01 vs. ovCON+TNF (one-way ANOVA). **i** Insulin signaling examined by western blotting analysis in 3T3-L1 adipocytes. Cells treated with TNF-α (4 ng/ml) alone or together with shMTPα. Meanwhile all adipocytes were treated with insulin (5 μg/ml) for 4 days. *p < 0.05, **p < 0.01, ***p < 0.001, ****p < 0.0001 vs. shCON; ^#^*p* < 0.05, ^##^*p* < 0.01, ^###^*p* < 0.001 vs. shCON+TNF (one-way ANOVA). Data represent at least three different experiments. *n* = 5 mice per group. All data represent mean ± standard error (SE).

We then examined MTPα expression in the IR model in 3T3-L1 adipocytes. Protein expression of MTPα was reduced in TNF-α-treated 3T3-L1 adipocytes, whereas mRNA expression was not significantly altered (Fig. 2a, b). We next sought to extend these observations to an in vivo IR model, using mice with high fat diet (HFD)-induced IR and db/db mice deficient for the leptin receptor. Results showed that MTPα protein levels were significantly lower in white adipose tissue (WAT) of HFD-induced IR mice (Fig. 2c) and db/db diabetic mice (Fig. 2d) than in control mice. However, mRNA expression was not significantly altered (Fig. 2a).

Next, we evaluated the role of MTPα in IR. We transfected 3T3-L1 preadipocytes with a lentiviral system that induced MTPα overexpression (ovMTPα). Western blotting showed about 50% transfection efficiency of ovMTPα in 3T3-L1 preadipocytes (Fig. 2e). OvMTPα attenuated TNF-α-induced IR, as evidenced by a dramatical increase in insulin-stimulated glucose uptake in TNF-α-treated 3T3-L1 adipocytes (*p* < 0.01, Fig. 2g). Further, ovMTPα in TNF-α treated 3T3-L1 adipocytes completely blocked the effect of TNF-α and enhanced insulin-stimulated IRS1 and Akt phosphorylation (Fig. 2h).

We also transfected 3T3-L1 preadipocytes with a shRNA lentiviral vector targeting MTPα mRNA (shMTPα), which stably knocked down MTPα expression in 3T3-L1 preadipocytes. Western blotting confirmed about 80% knockdown efficiency of MTPα (Fig. 2f). MTPα knockdown enhanced TNF-α-induced insulin resistance, as evidenced by significantly decreased insulin-stimulated glucose uptake in TNF-α-treated 3T3-L1 adipocytes (*p* < 0.0001, Fig. 2g). Moreover, shMTPα treatment further decreased insulin-stimulated phosphorylation of IRS1 and Akt in TNF-α treated 3T3-L1 adipocytes (Fig. 2i). Taken together, these data indicate that MTPα overexpression reduced IR induced by TNF-α, whereas MTPα knockdown increased IR.

### MTPα promoted insulin-stimulated Glut4 translocation to the plasma membrane in 3T3-L1 adipocytes

**Fig. 3:**
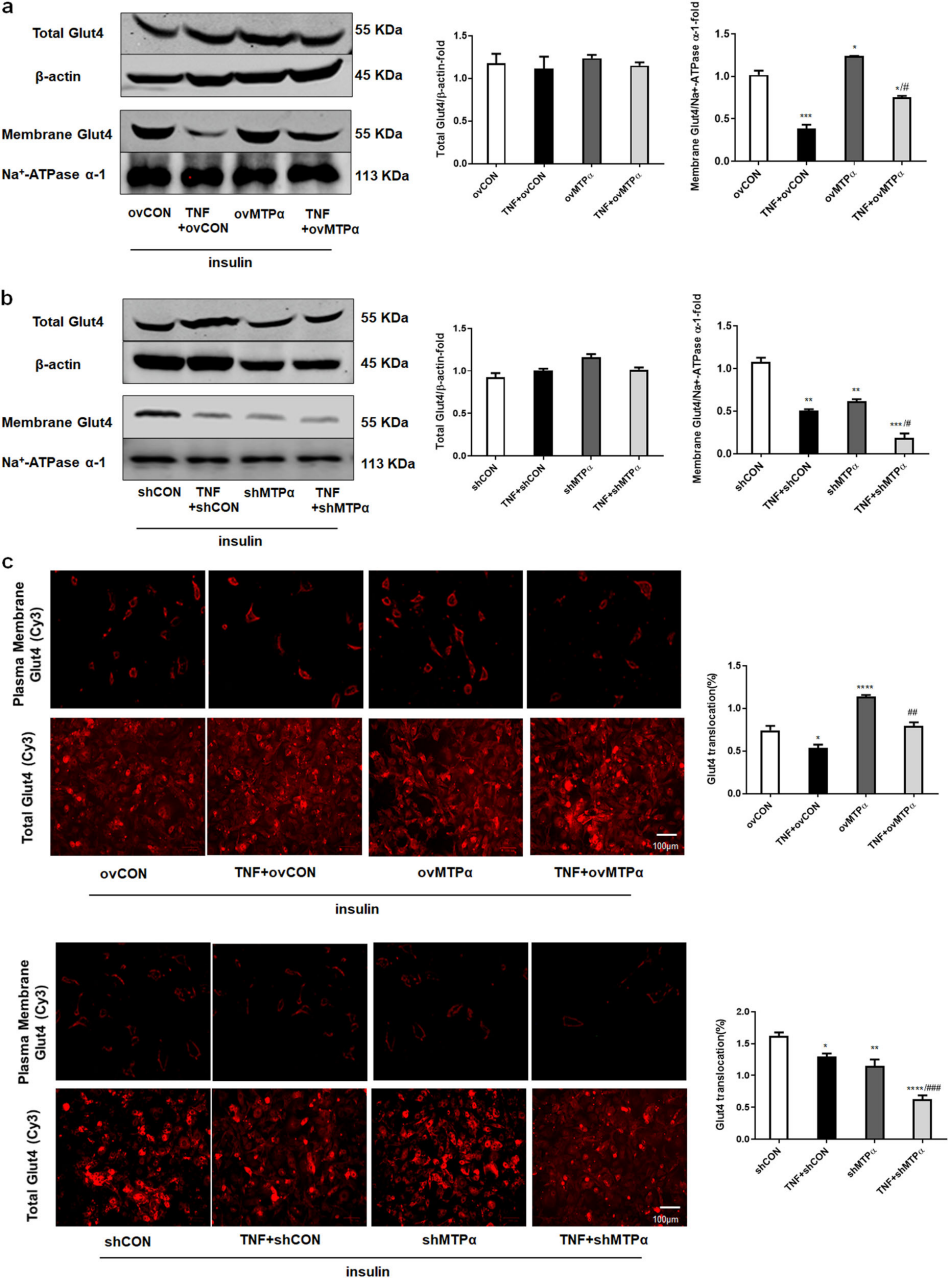
MTPα promoted insulin stimulated Glut4 translocation in 3T3-L1 adipocytes. **a** Total Glut4 protein levels and plasma membrane Glut4 protein levels were measured by western blotting in 3T3-L1 adipocytes transfected with control siRNA or ovMTPα, treated with or without TNF-α (4 ng/ml) for 4 days. Meanwhile all adipocytes were treated with insulin (5 μg/ml) for 4 days. *p < 0.05, ***p < 0.001 vs. ovCON; ^#^*p* < 0.05 vs. TNF+ ovCON (one-way ANOVA). **b** Total Glut4 protein levels and plasma membrane Glut4 protein levels were measured by western blotting in 3T3-L1 adipocytes transfected with control siRNA or shMTPα, treated with or without TNF-α (4 ng/ml) for 4 days. Meanwhile insulin (5 μg/ml) was added into media. **p < 0.05, ***p < 0.001 vs. shCON; ^#^*p* < 0.05 vs. TNF + shCON (one-way ANOVA). **c** Immunofluorescence detection of Glut4 plasma membrane translocation in 3T3-L1 adipocytes transfected with control siRNA, shMTPα or ovMTPα and treated with or without TNF-α (4 ng/ml) for 4 days. Meanwhile insulin (5 μg/ml) was added into media. Scale bar: 100 μm. *p < 0.05, **p < 0.01, ***p < 0.001, ****p < 0.0001 vs. ovCON or shCON; ^##^*p* < 0.01, ^###^*p* < 0.001 vs. TNF + ovCON or TNF + shCON (one-way ANOVA). Data represent at least three different experiments. All data represent means ± standard error (SE).

To further determine how glucose uptake was enhanced by MTPα, we analyzed MTPα effects on Glut4 translocation to the plasma membrane in 3T3-L1 adipocytes. TNF-α treatment alone or together with shMTPα or ovMTPα had no effect on total Glut4 expression in 3T3-L1 adipocytes (Fig. 3a, b). However, TNF-α treatment decreased insulin-stimulated Glut4 protein levels in the plasma membrane of 3T3-L1 adipocytes compared to controls. MTPα overexpression increased insulin-stimulated Glut4 protein levels to nearly baseline levels. In contrast, MTPα knockdown further decreased insulin-stimulated Glut4 protein levels in the plasma membrane of TNF-α -treated 3T3-L1 adipocytes (Fig. 3a–c). These results suggest that MTPα promoted Glut4 translocation.

### SIRT1 reversed IR via an MTPα-related pathway

**Fig. 4:**
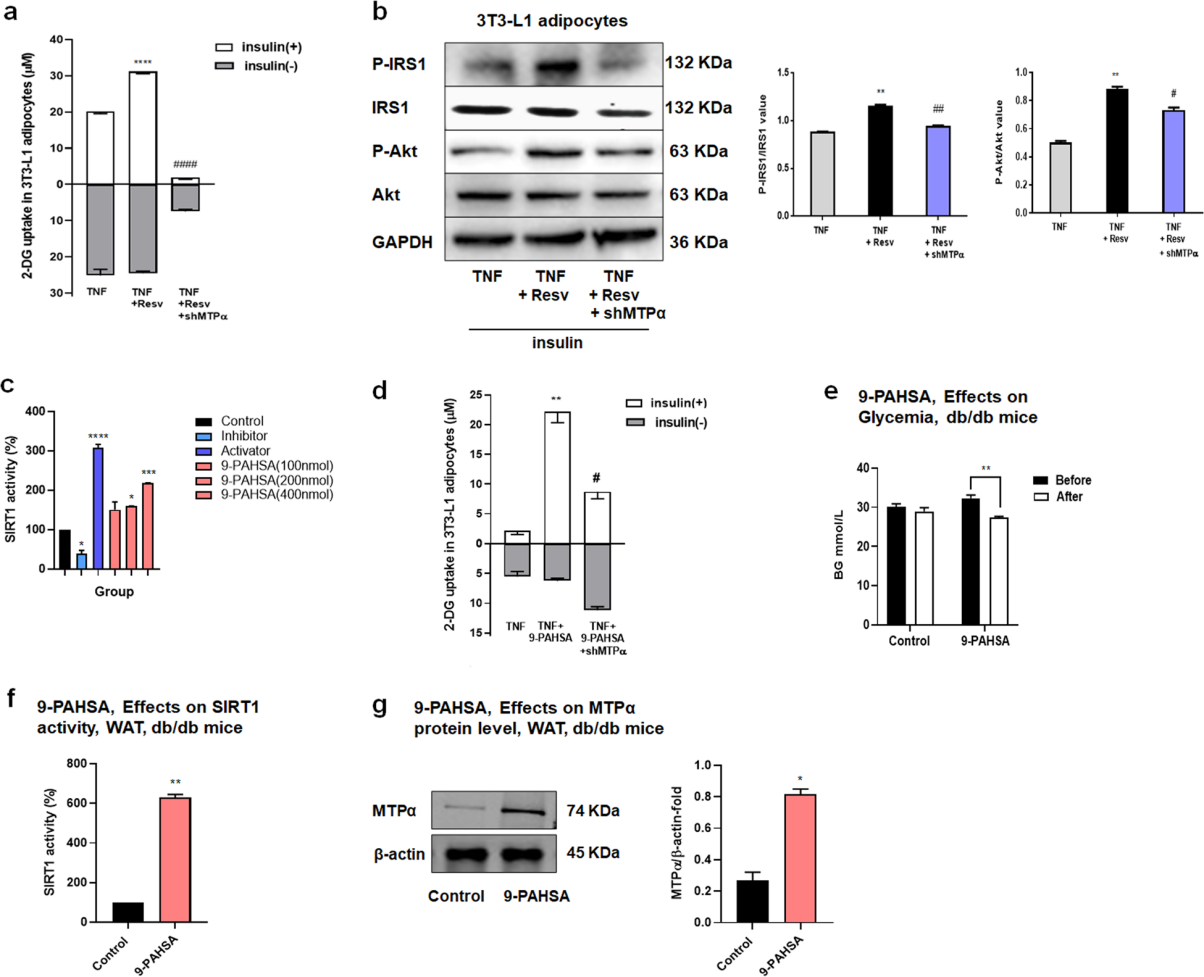
SIRT1 reversed insulin resistance via an MTPα-related pathway. **a** Glucose uptake in TNF-α-induced insulin-resistant 3T3-L1 adipocytes transfected with control siRNA or shMTPα and treated with resveratrol (10 μM). Basal glucose transport (gray) and insulin stimulated glucose uptake (white) are shown. ****p < 0.0001 vs. TNF plus insulin; ^####^*p* < 0.0001 vs. TNF + Resv plus insulin (one-way ANOVA). **b** Insulin signaling measured by western blotting analysis in 3T3-L1 adipocytes. 3T3-L1 adipocytes transfected with control siRNA or shMTPα were treated with TNF-α (4 ng/ml) alone or together with resveratrol (10 μM) during the induction of insulin resistance. All adipocytes were treated with insulin (5 μg/ml) for 4 days. **p < 0.01 vs. TNF; ^#^*p* < 0.05, ^##^*p* < 0.01 vs. TNF + Resv (one-way ANOVA). **c** SIRT1 activity determined by SIRT1 fluorometric assay kit for activator screening. 9-PAHSA at different concentrations (100 nmol, 200 nmol, 400 nmol) was incubated with SIRT1 enzyme, SIRT1 substrate, and NAD+ in vitro. An inhibitor (nicotinamide) and an activator (resveratrol) were added as negative and positive controls, respectively. *p < 0.05, ****p < 0.0001 vs. control (one-way ANOVA). **d** Glucose uptake in TNF-α-insulin-resistant 3T3-L1 adipocytes transfected with control siRNA or shMTPα and treated with 9-PAHSA (20 μM) during the induction of insulin resistance. Basal glucose transport (gray) and insulin stimulated glucose uptake (white) are shown. **p < 0.01 vs. TNF plus insulin; ^#^*p* < 0.05 vs. TNF + 9-PAHSA plus insulin (one-way ANOVA). **e** Glycemic level in db/db mice after 10 days 9-PAHSA administration (50 mg/kg) in db/db mice. **p < 0.01 (*t*-test). **f** SIRT1 activity in WAT of db/db mice after 10 days 9-PAHSA administration (50 mg/kg). **p < 0.01 vs. control (*t*-test). **g** MTPα protein expression in WAT of db/db mice after 10 days 9-PAHSA administration (50 mg/kg). *p < 0.05 vs. control (*t*-test). Data represent at least three different experiments. *n* = 5 mice per group. All data represent means ± standard error (SE).

Resveratrol activates SIRT1 to reverse insulin resistance^19^. To investigate the mechanism underlying SIRT1 anti-diabetic activity, we added the SIRT1 activator resveratrol to TNF-α-induced insulin-resistant adipocytes. Resveratrol increased insulin-stimulated glucose uptake in TNF-α-induced insulin-resistant 3T3-L1 adipocytes (Fig. 4a). We performed the same experiment in 3T3-L1 adipocytes with MTPα-knockdown. The results showed that shMTPα prevented the resveratrol-induced increase in insulin-dependent glucose uptake. We also examined how resveratrol and shMTPα affected insulin signaling, finding that shMTPα decreased the resveratrol-induced insulin stimulated phosphorylation of Akt and IRS1 (Fig. 4b). These results indicate that resveratrol reduced IR via an MTPα-related pathway.

To confirm whether SIRT1 reversed IR through MTPα, we used 9-PAHSA, a novel activator of SIRT1 that is an endogenous mammalian lipid with anti-diabetic effects^20^. We found that 9-PAHSA significantly increased SIRT1 activity in a dose-dependent manner (Fig. 4c). Further, 9-PAHSA treatment in 3T3-L1 adipocytes increased SIRT1 activity, similar to the effect of resveratrol treatment (Supplementary Fig. S1). Also similar to resveratrol, 9-PAHSA increased insulin-stimulated glucose uptake in insulin-resistant 3T3-L1 adipocytes. However, this action of 9-PAHSA was reversed by shMTPα (Fig. 4d). Notably, oral administration of 9-PAHSA in db/db mice lowered basal glycemia (Fig. 4e). Mechanistically, 9-PAHSA administration increased SIRT1 activation and increased MTPα protein levels in WAT of db/db mice (Fig. 4f, g).

### SIRT1 increased MTPα protein levels by inhibiting MTPα ubiquitylation

**Fig. 5:**
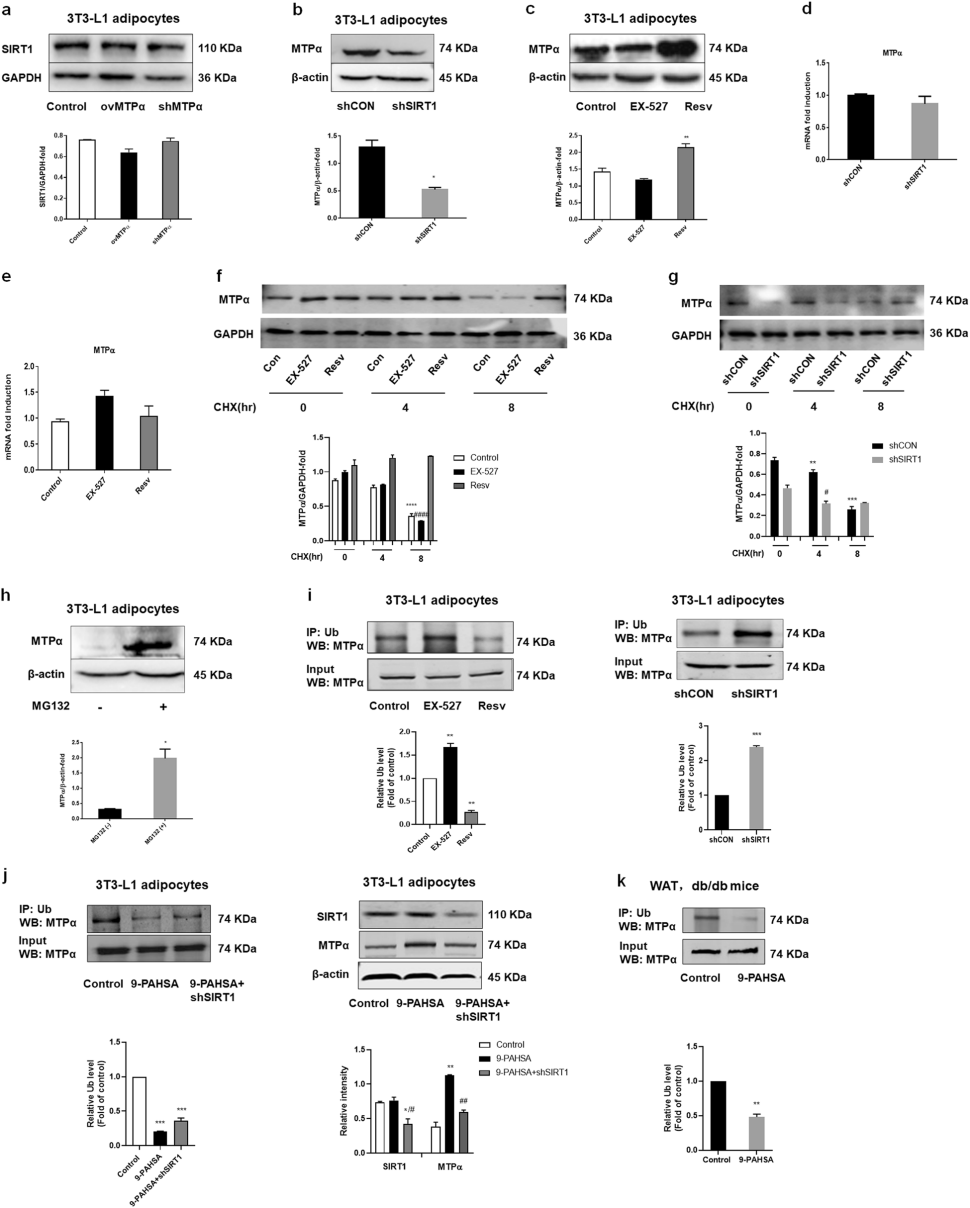
SIRT1 increased MTPα protein levels by inhibiting MTPα ubiquitylation. **a** SIRT1 protein levels in 3T3-L1 adipocytes transfected with control siRNA, ovMTPα, or shMTPα. **b** MTPα protein levels in 3T3-L1 adipocytes transfected with control siRNA or shSIRT1. *p < 0.05 vs. shCON (*t*-test). **c** MTPα protein levels in 3T3-L1 adipocytes treated with EX-527 (10 μM) or resveratrol (10 μM) for 4 days. **p < 0.01 vs. control (one-way ANOVA). **d** MTPα mRNA levels in 3T3-L1 adipocytes transfected with control siRNA or shSIRT1. **e** MTPα mRNA levels in 3T3-L1 adipocytes treated with EX-527 (10 μM) or resveratrol (10 μM) for 4 days. **f** 3T3-L1 adipocytes treated with EX-527 (10 μM) or resveratrol (10 μM) for 4 days, then treated with CHX for the indicated time and subjected to western blotting and quantified. ***p < 0.001 vs. control (CHX 0); ^####^*p* < 0.001 vs. EX-527 (CHX 0) (two-way ANOVA). **g** 3T3-L1 adipocytes transfected with control siRNA or shSIRT1, treated with CHX for the indicated time and subjected to western blotting and quantified. **p < 0.01, ***p < 0.001 vs. control (CHX 0); ^#^*p* < 0.001 vs. shSIRT1 (CHX 0) (two-way ANOVA). **h** MTPα protein levels in 3T3-L1 adipocytes treated with (+) or without (−) MG132 (10 μM) for 4 h. *p < 0.05 vs. MG132 (−) (*t*-test). **i** 3T3-L1 adipocytes treated with EX-527 (10 μM) or resveratrol (10 μM) for 4 days, or transfected with control siRNA or shSIRT1. Then MTPα was immunoprecipitated with anti-Ub antibody from 3T3-L1 adipocytes and ubiquitylation levels assessed by western blotting. **p < 0.01 vs. control (one-way ANOVA). ***p < 0.001 vs. shCON (*t*-test). **j** MTPα ubiquitylation and protein expression of SIRT1 and MTPα were measured in 3T3-L1 adipocytes transfected with control siRNA or shSIRT1 and treated with 9-PAHSA (20 μM) for 4 days. *p < 0.05, **p < 0.01, ***p < 0.001 vs. control, ^#^*p* < 0.05, ^##^*p* < 0.01 vs. 9-PAHSA (one-way ANOVA). **k** MTPα ubiquitylation measured in WAT of db/db mice after 10 days 9-PAHSA administration (50 mg/kg). **p < 0.01 vs. control (t test). Data represent at least three different experiments. *n* = 5 mice per group. All data represent means ± standard error (SE).

To investigate interactions between MTPα and SIRT1, we first examined SIRT1 expression in 3T3-L1 adipocytes transfected with shMTPα or ovMTPα. Knockdown or overexpression of MTPα had no effect on SIRT1 protein levels (Fig. 5a). We then examined MTPα expression in 3T3-L1 adipocytes transfected with shSIRT1. The results showed that SIRT1 knockdown decreased MTPα protein expression (Fig. 5b). Treatment of EX527, the SIRT1-specific inhibitor, in 3T3-L1 adipocytes also decreased MTPα protein expression, although the change was not significant. Moreover, resveratrol, the SIRT1 activator, increased MTPα protein expression (Fig. 5c). Considering resveratrol also activated the mitochondrial sirtuin, SIRT3, we tested the the role of resveratrol in MTPα protein expression by knocking down SIRT3. The results showed that resveratrol treatment also increased MTPα protein expression in SIRT3 knockdown cells (Supplementary Fig. S2), further indicating a specific interaction between SIRT1and MTPα. Notably, MTPα mRNA levels were not affected by shSIRT1, resveratrol or EX-527 treatment (Fig. 5d, e). It is indicated that SIRT1 may regulate MTPα by altering post-translational modification.

In first, we investigated and observed a physical interaction between SIRT1and MTPα. It is acknowledged that MTPα localizes to the mitochondrial. SIRT1 is also found in mitochondria using confocal microscopy (Supplementary Fig. S3a). Protein extracts of purified mitochondria from adipocytes further displayed the presence of SIRT1 in mitochondria (Supplementary Fig. S3b).

We then explored the mechanism underlying SIRT1-mediated upregulation of MTPα. Our results showed that the proteasomal inhibitor MG132 significantly increased MTPα protein levels (*p* < 0.05, Fig. 5h), indicating that the ubiquitin proteasome pathway degrades MTPα. Inhibition of protein synthesis with cycloheximide (CHX) showed that MTPα is an unstable protein with a half life of about 8 h. Activating SIRT1 by resveratrol substantially extended the half life of MTPα, while blocking SIRT1 activity by EX-527 or SIRT1 knockdown had adverse effects (Fig. 5f, g). Our data further demonstrate that EX527 treatment increased MTPα ubiquitylation, but resveratrol treatment decreased it (Fig. 5i). Further, SIRT1 knockdown increased MTPα ubiquitylation (Fig. 5i).

To confirm these findings, we also measured MTPα ubiquitylation in 9-PAHSA-treated 3T3-L1 adipocytes and in WAT from 9-PAHSA-treated db/db mice. Similarly, 9-PAHSA treatment suppressed MTPα ubiquitylation and increased MTPα protein levels. However, shSIRT1 decreased this effect of 9-PAHSA treatment (Fig. 5j, k). Taken together, these data suggest that SIRT1 repressed MTPα ubiquitylation and subsequent degradation.

### SIRT1 mediated MTPα deacetylation, thus inhibiting MTPα ubiquitylation and reducing IR

**Fig. 6:**
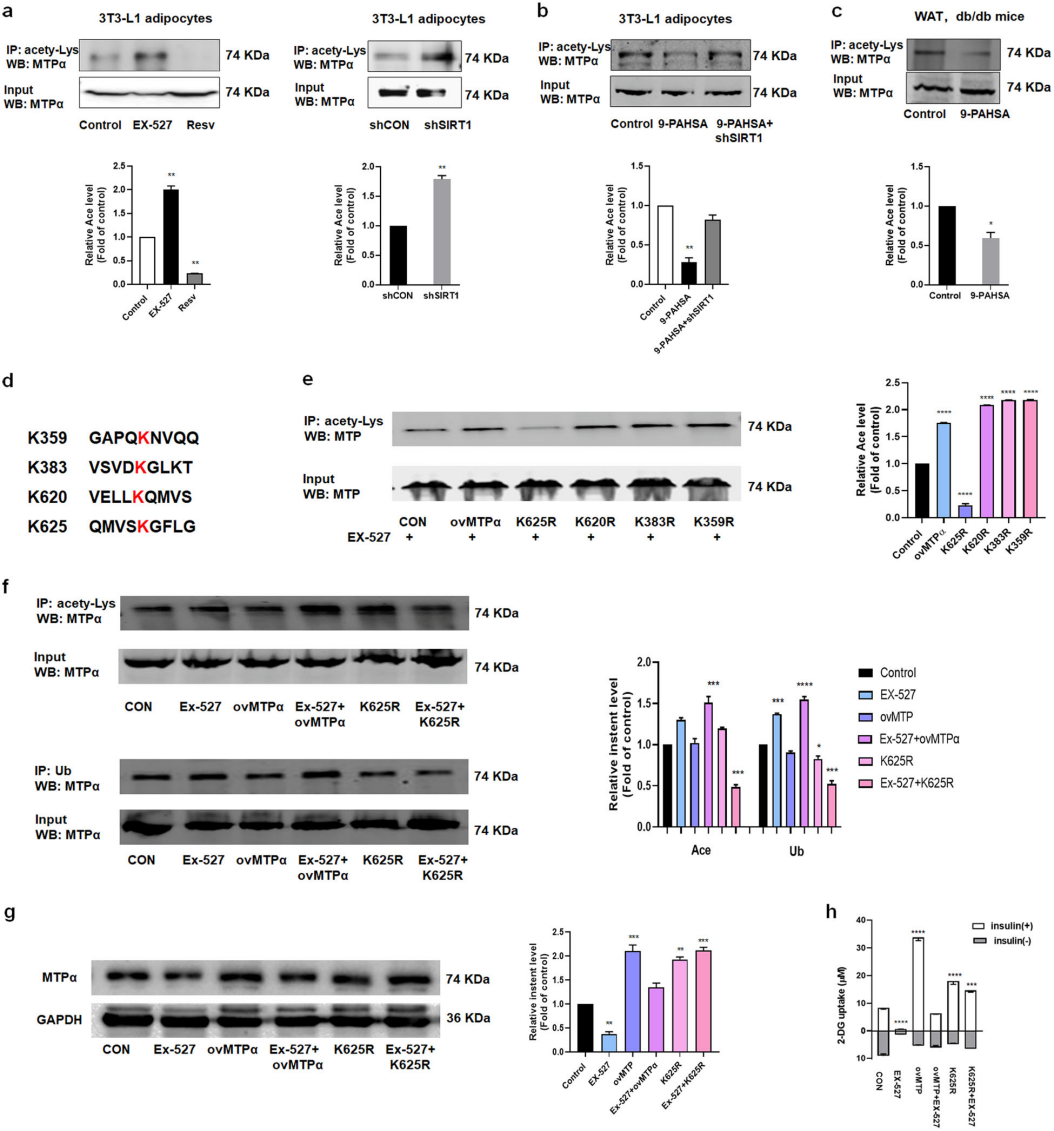
SIRT1 deacetylated MTPα, inhibited MTPα ubiquitylation, and reduced IR. **a** 3T3-L1 adipocytes treated with EX-527 (10 μM) or resveratrol (10 μM) for 4 days, or transfected with control siRNA or shSIRT1. Then MTPα was immunoprecipitated with anti-acetyl lysine from 3T3-L1 adipocytes and acetylation levels were assessed by western blotting. **p < 0.01 vs. control (one-way ANOVA). **p < 0.01 vs. shCON (*t-*test). **b** MTPα acetylation measured in 3T3-L1 adipocytes treated with 9-PAHSA (20 μM) and transfected with shSIRT1. **p < 0.01 vs. control (one-way ANOVA). **c** MTPα acetylation measured in WAT of 9-PAHSA (50 mg/kg) treated db/db mice. *p < 0.05 vs. control (*t*-test). **d** MTPα acetylation sites detected by MS spectrometry of 3T3-L1 adipocytes. **e** MTPα acetylation measured in 3T3-L1 adipocytes transfected with ovMTPα or lysine mutants and then treated with EX-527 (10 μM) for 4 days. ****p < 0.0001 vs. control (one-way ANOVA). **f** MTPα acetylation and ubiquitylation measured in 3T3-L1 adipocytes transfected with ovMTPα or K625R and then treated with EX-527 (10 μM) for 4 days. *p < 0.05, ***p < 0.001, ****p < 0.0001 vs. control (one-way ANOVA). **g** MTPα protein level measured in 3T3-L1 adipocytes transfected with ovMTPα or K625R and then treated with EX-527 (10 μM) for 4 days. **p < 0.01, ***p < 0.001 vs. control (one-way ANOVA). **h** Glucose uptake in TNF-α-induced insulin-resistant 3T3-L1 adipocytes transfected with K625R or ovMTPα, and/or treated with EX-527 (10 μM) for 4 days. Basal glucose transport (gray) and insulin stimulated glucose uptake (white) are shown. ***p < 0.001, ****p < 0.0001 vs. TNF plus insulin (CON) (one-way ANOVA). Data represent at least three different experiments. *n* = 5 mice per group. All data represent means ± standard error (SE).

SIRT1 has deacetylation enzymatic activity. To identify posttranslational modifications of MTPα regulated by SIRT1, we measured MTPα acetylation after immunoprecipitating MTPα and western blotting with anti-acetyl lysine antibody. We found that EX527 treatment increased MTPα acetylation and resveratrol treatment decreased MTPα acetylation, relative to controls (Fig. 6a). We also detected more MTPα acetylation in shSIRT1 transfected 3T3-L1 adipocytes than in shCON adipocytes (Fig. 6a). Furthermore, 9-PAHSA treatment decreased MTPα acetylation in both 3T3-L1 adipocytes and WAT of db/db mice. However, shSIRT1 reversed this decreased MTPα acetylation in 3T3-L1 adipocytes (Fig. 6b, c). These data demonstrate that SIRT1 regulated MTPα acetylation.

To identify MTPα acetylation sites, we conducted mass spectrometry analysis on 3T3-L1 adipocytes. We identified four acetylation sites on MTPα (Fig. 6d). To determine which MTPα acetylation sites were regulated by SIRT1, we mutated each candidate lysine to an arginine (R). We transfected 3T3-L1 adipocytes with wild type, ovMTPα (positive control), or MTPα mutants, treated cells with EX-527, and then performed immunoprecipitation. K625 mutation greatly decreased MTPα acetylation, whereas mutation of K383, K620 or K359 had no effect on MTPα acetylation (Fig. 6e). In addition, MTPα acetylation was higher in ovMTPα transfected adipocytes treated with EX-527 than in untreated cells. However, EX-527 treatment did not further increase MTPα acetylation when the K625 site was mutated (Fig. 6f), suggesting that K625 is the MTPα acetylation site regulated by SIRT1.

We also found that SIRT1-mediated MTPα acetylation affected MTPα ubiquitylation. As shown in Fig. 5, inhibiting SIRT1 deacetylases with EX-527 increased MTPα ubiquitylation. Further, activating SIRT1 deacetylases with resveratrol or 9-PAHSA reduced MTPα ubiquitylation. This finding led us to investigate potential crosstalk between MTPα acetylation and ubiquitylation. We found that K625 mutation decreased MTPα ubiquitylation levels. Furthermore, EX-527 increased ovMTPα-induced ubiquitylation, but did not increase K625R ubiquitylation (Fig. 6f). Besides, K625 mutation blocked the degradation of MTPα. Moreover, treatment with EX-527 could not further decrease the protein levels of K625 mutation (Fig. 6g). Our results suggest that acetylation of the K625 lysine site promoted MTPα ubiquitylation and degradation.

We found that K625 mutation, much like ovMTPα, increased insulin-stimulated glucose uptake in TNF-α-induced insulin-resistant cells, indicating that K625R improved IR. Moreover, EX-527 did not attenuate the effect of K625R mutation (Fig. 6h), suggesting that SIRT1 decreased IR by inhibiting MTPα ubiquitylation.

## Discussion

MTPα is an important enzyme involved in FAO. Defective FAO produces excess free fatty acids, contributing to the development of IR. In this study, we identified a new function for MTPα in glucose metabolism. We found that MTPα expression was significantly decreased in insulin-resistant cell models and diabetic mice. Importantly, MTPα overexpression reduced IR. In contrast, MTPα down-regulation promoted IR. We found that SIRT1 mediated this activity by deacetylating MTPα and inhibiting MTPα degradation.

Previous studies show that lysine acetylation is an important mechanism for regulating glucose homeostasis^21,22^. Our findings support this previous work with the discovery that MTPα acetylation is a regulatory mechanism underlying IR. We identified four lysine sites (K383, K620, K359, and K625) on MTPα in insulin-resistant 3T3-L1 adipocytes. SIRT1 reportedly protects against diet-induced insulin resistance^23^ and enhances insulin signaling in multiple types of insulin sensitive cells^24^. In our study, SIRT1 deacetylated MTPα at the K625 site. Moreover, SIRT1 regulation of MTPα increased glucose uptake by deacetylating K625 on MTPα. From these findings, we conclude that SIRT1-regulated MTPα deacetylation is crucial to maintain glucose homeostasis. Deacetylation and acetylation are known to affect enzyme activity. For example, deacetylation of trifunctional enzyme subunit alpha (ECHA) promotes FAO^25^. Lactate dehydrogenase A is acetylated at K5 and this acetylation inhibits its enzymatic activity^26^. SIRT5-mediated deacetylation of carbamoyl phosphate synthetase 1 increases its amino acid catabolism activity^27^. Hyperglycemia contributes to hyperacetylation of fatty acid β-oxidation enzymes, resulting in dysregulated energy metabolism^28^. Similar to these previous reports, we found that deacetylation of MTPα reduced IR. Our study showed that IR downregulated MTPα. SIRT1 activation also affected MTPα protein levels, but not MTPα mRNA expression. Specifically, EX-527 treatment decreased MTPα protein expression, whereas resveratrol increased MTPα protein levels. This leads us to hypothesize that SIRT1 regulates MTPα degradation by regulating MTPα acetylation.

A previous study reported that a ubiquitin proteasome pathway degrades MTPα^14^. The ubiquitin-proteasome system (UPS) is important for controlling levels of various cellular proteins and regulating degradation of mitochondrial proteins. We consistently found that treating 3T3-L1 adipocytes with the proteasomal inhibitor MG132 increased MTPα protein levels, suggesting that MTPα is a target of UPS. Studies report^14^ that MTPα acetylation prevents its ubiquitylation, suggesting that acetylation and ubiquitylation in MTPα may compete with each other by targeting the same lysine residues. However, we found that acetylation of MTPα promoted its ubiquitylation. The difference between our study and previous results may be due to different cell and insulin-resistant models. In our study, EX-527 increased MTPα ubiquitylation by inhibiting MTPα deacetylation in insulin-resistant 3T3-L1 adipocytes. K625 site mutation disrupted MTPα acetylation and ubiquitylation, and EX-527 treatment did not restore these modifications. Thus, our results support the idea that MTPα acetylation promoted its ubiquitylation. Besides, the lysine acetylation sites on MTP between the two studies are different. Liang Guo, et al.^14^ show that MTPα is acetylated at lysine residues 350/383/406 (MTPα-3K), which promotes its protein stability by antagonizing its ubiquitylation on the three same lysines (MTPα-3K) and blocking its subsequent degradation. SIRT4 deacetylated MTPα at lysine residues 350/383/406 and destabilized it. In our study, we identified four lysine-acetylated sites (K383, K620, K359, and K625) on MTPα by MS. SIRT1 deacetylated MTPα at the K625 site and repressed its degradation. Consistent with our findings, acetylation reportedly decreased ECHA protein stability, due to SIRT3 overexpression preventing ECHA degradation by decreasing its acetylation in β-cells^25^.

SIRT1 is a metabolic sensor with many roles in regulating cell biology, such as energy metabolism. However, the mechanism behind SIRT1 activity is not fully understood although it is known that SIRT1 NAD-dependent deacetylase activity mediates many of its functions. In our study, we found that SIRT1 also localized in mitochondria in addition to the cytoplasm and the nucleus. SIRT1-mediated deacetylation of MTPα at K625 prevented MTPα ubiquitylation. Moreover, SIRT1 knockdown in 3T3-L1 adipocytes downregulated MTPα protein levels. This finding was consistent with previous studies that showed that decreased SIRT1 expression caused lower MTPα protein expression^29^. We also found that K625 mutation enhanced insulin-dependent glucose uptake and reduced IR caused by TNF-α treatment. Together, these findings indicate that SIRT1 reduced IR by deacetylating MTPα and inhibiting MTPα degradation.

Resveratrol, a natural phenol found in the skin of grapes and blueberries, is a SIRT1 activator. Reportedly, resveratrol improves glucose control and insulin sensitivity in animal and cell culture studies^19^. However, the efficacy and safety of resveratrol treatment in humans requires further study. An alternative to resveratrol, 9-PAHSA, may activate SIRT1 and reduce MTPα acetylation. Notably, 9-PAHSA is an endogenous fatty acid that reduces blood glucose levels and inflammation^20^. Thus, 9-PAHSA is a promising candidate for further therapeutic investigation.

**Fig. 7:**
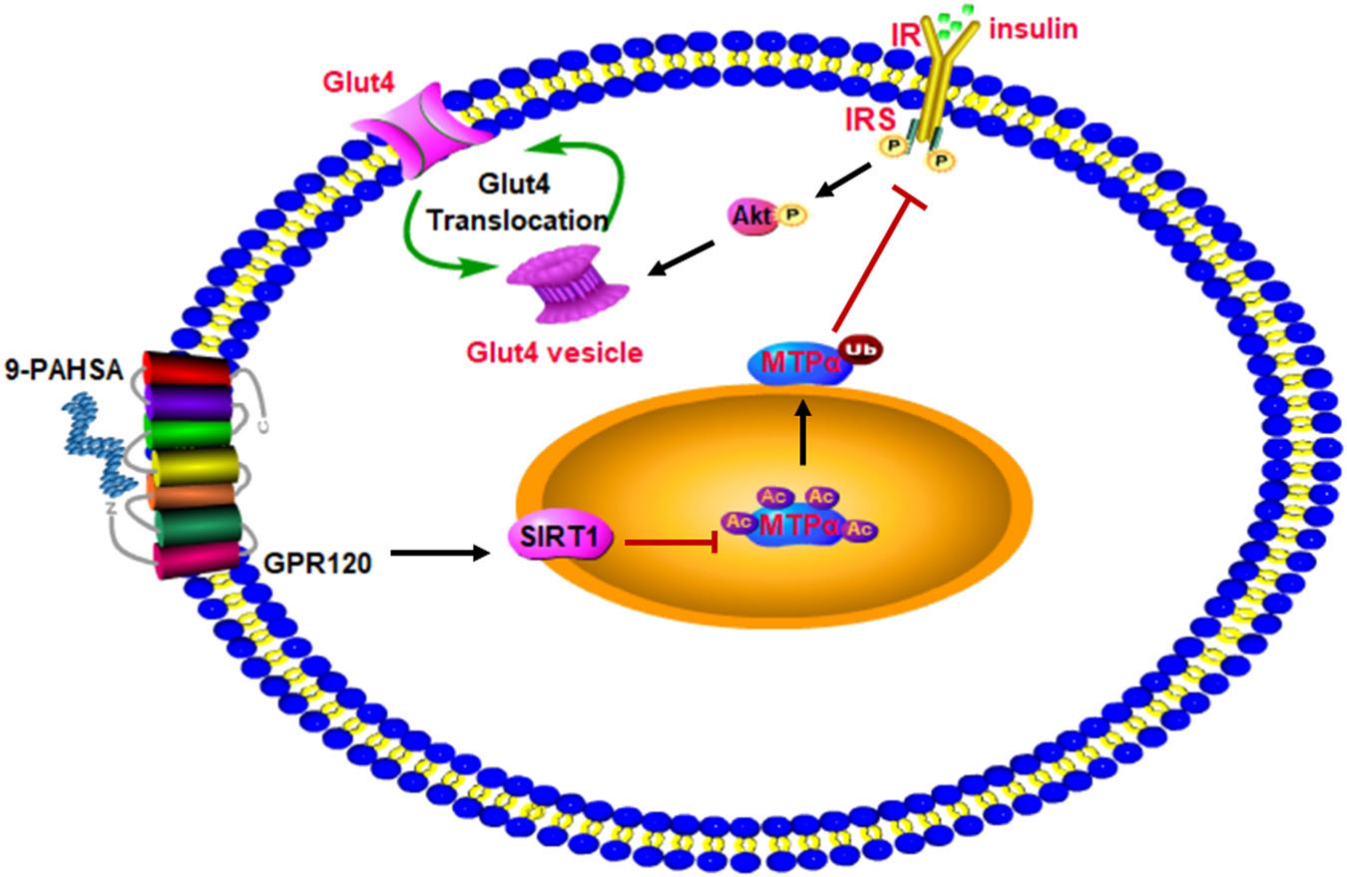
Deacetylation of MTPα mediated by SIRT1 inhibited MTPα ubiquitylation and reduced insulin resistance. SIRT1 activation by 9-PAHSA decreased acetylation and ubiquitylation of MTPα, which inhibited MTPα degradation and activated the insulin signaling pathway.

In summary, we present a novel function for MTPα in reducing IR. The modification of MTPα by acetylation and ubiquitylation was crucial for regulating MTPα function in glucose metabolism (Fig. 7). SIRT1, a mitochondrial deacetylase, reduced MTPα acetylation and prevented MTPα degradation, resulting in increased insulin dependent glucose uptake in insulin-resistant cells. Therefore, 9-PAHSA, a SIRT1 activator, is a new and promising means to reduce IR.

## Supplementary information

supplementary figure legend

Supplementary Figure 1

Supplementary Figure 2

Supplementary Figure 3
