# Supplementary material for: SIRT1 deacetylates mitochondrial trifunctional enzyme α subunit to inhibit ubiquitylation and decrease insulin resistance
Source: Cell Death Dis. 2020 Oct 2;11(10):821. doi: 10.1038/s41419-020-03012-9 (PMC7532168; doi:10.1038/s41419-020-03012-9)
Supplement: Supplementary file 1 — supplementary figure legend [file 41419_2020_3012_MOESM1_ESM.docx]

**Supplementary data**

**Supplement 1. The SIRT1 activity in 3T3-L1 adipocytes.** The SIRT1 activity was determined using a SIRT1 fluorometric assay kit. 3T3-L1 adipocytes were treated with 9-PAHSA、EX527 and resveratrol. *p<0.05 versus control (one-way ANOVA). Data are representative of at least three different experiments. All data represent means ± standard error (SE).

**Supplement 2. The effect of resveratrol on** **MTPα protein expression by knocking down SIRT3.** (a) The protein expression of SIRT3 in 3T3-L1 cells transfected with control siRNA (CON siRNA) or SIRT3 siRNA. ***p < 0.001 versus CON siRNA (t test). (b) Resveratrol treatment increased MTPα protein expression in SIRT3 knockdown cells. *p < 0.05 versus control (t test). Data are representative of at least three different experiments. All data represent means ± standard error (SE).

**Supplement 3. The subcellular localization of SIRT1 in 3T3-L1 cells.** (a) SIRT1 (red) expressed in 3T3-L1 cells was examined by immunofluorescence using SIRT antibody and the mitochondrial marker Mitotracker (green). DAPI was used to stain the cell nuclei (blue). (b) Proteins obtained from 3T3-L1 adipocytes purified mitochondria (Mito) and intact adipocytes (Total) were subjected to SDS-PAGE followed by Western blot analysis. The effective mitochondria isolation was assessed by anti-VDAC1. The possible presence of nuclear protein contaminants was measured by anti-H2B.
