# Supplementary figures and images for: SIRT1 deacetylates mitochondrial trifunctional enzyme α subunit to inhibit ubiquitylation and decrease insulin resistance
Source: Cell Death Dis. 2020 Oct 2;11(10):821. doi: 10.1038/s41419-020-03012-9 (PMC7532168; doi:10.1038/s41419-020-03012-9)

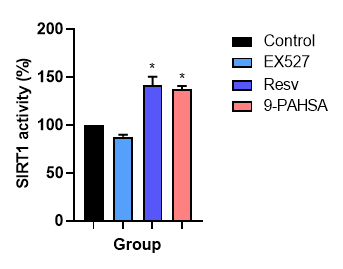

Supplement: Supplementary file 2 — Supplementary Figure 1 [file 41419_2020_3012_MOESM2_ESM.tif]

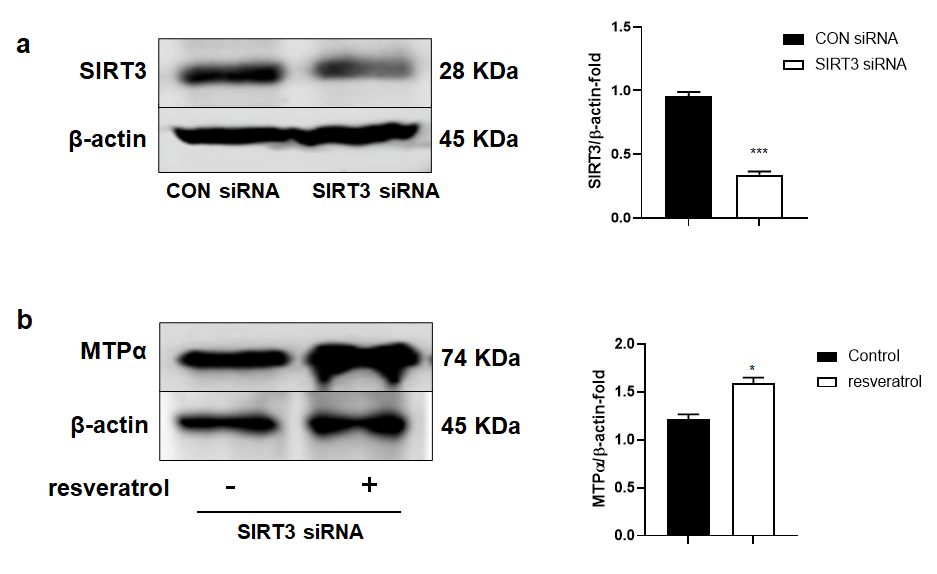

Supplement: Supplementary file 3 — Supplementary Figure 2 [file 41419_2020_3012_MOESM3_ESM.tif]

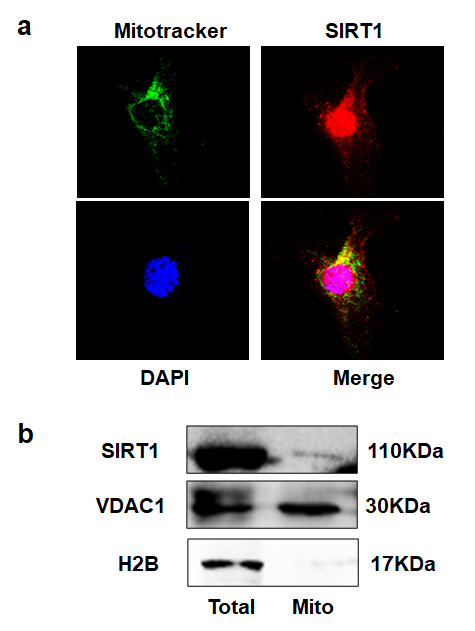

Supplement: Supplementary file 4 — Supplementary Figure 3 [file 41419_2020_3012_MOESM4_ESM.tif]
